# National Guard or Continental Army?

**Published:** 1916-02

**Authors:** 


### National Guard or Continental Army?

Our brief allusion to this problem has aroused considerable interest and one of the members of the medical staff of the local Guard, has sent a reprint of the address of Maj. Gen. John F. O'Ryan, commanding N. Y. Div. before the Convention of the National Guard Assn. at San Francisco, Nov. 11. He presents both sides fairly but holds that the National Guard should be developed, rather than a new army instituted, because the former has quarters and equipment, can give training throughout the year with only the ordinary business vacation in the field, can keep track of members and, in an emergency, more rapidly assemble retired soldiers and new recruits.

These advantages have a special application to the medical profession. War on such a scale as is now being waged in Europe, would require the services of nearly every physician of proper age and physical fitness. In a sense, the medical staff of an army does not need military training. That is to say, actual professional duties are either part of the experience of the individual doctor or must be learned according to actual conditions which vary for different wars and whose management is in accordance with well-known general principles. What may be called red-tape, can be acquired by men of the average intelligence and education of the medical profession, very quickly. Strictly military precedents are not of great practical importance and strictly military procedures are beyond the province of the surgeon. But, in another sense, not one of these statements is true. It is highly desirable that the surgeon should have a reasonable comprehension of the duties of his associate officers and of the men who will be, in a sense, under his command, in a military as well as in a professional sense. Something more than aesthetics demands that he should be more than a civilian in uniform and with a commission.

Perhaps the problem can be best expressed in a concrete, local form. Such a war as is conceivable as a reason for military preparedness, would enlist 200 or possibly 300 physicians from Buffalo. The local National Guard at present numbers about 1500. If brought to its full quota of about 2500-3000, it would still afford positions for only 10 or 12 physicians. It would be impossible, except under the compulsion of absolute necessity, to train the large remainder of physicians in a Continental Army, intensively, in camp at a distance during a period of two or three months. But it would be perfectly feasible to establish schools for them, at fairly convenient times, during the year, under the auspices of the local regiments in their armories. The regular army officer is trained at West Point, as a private soldier, not because it is expected that he will subsequently serve as a private, but because he can best command and comprehend the psychology and conditions of the private from having had this experience. The same argument applies also to medical officers who should have, at least, the rudimentary drill of the private. It is out of the question for the average physician to absent himself from his work for any such period as is required for intensive camp instruction. In this particular, he is differently circumstanced from the analogous business man who actually spends very little time at his desk, even in his normal business life.

It occurs to us as very pertinent to inquire whether our local

National Guard has the authority and can arrange for classes in military training, general and with special reference to the needs of the military surgeon and how many physicians would, solely as volunteers and as a matter of patriotism, avail themselves of such classes. One somewhat delicate subordinate question presents itself: Suppose that an individual is beyond the established military age, but is habitually doing the work and enjoying the recreations of a much younger man; should age alone exempt him from his duty to his country: and supposing that he is personally willing to waive the age-exemption, will his country accept his services on the basis of his arteries, muscles and habits, or refuse them on the basis of the family Bible?

